# Prognostic role of neutrophil lymphocyte ratio in patients with spontaneous intracerebral hemorrhage

**DOI:** 10.18632/oncotarget.20776

**Published:** 2017-09-08

**Authors:** Jing Zhang, Linrui Cai, Yanlin Song, Baoyin Shan, Min He, Qingqing Ren, Chaoyue Chen, Zhiyong Liu, Yunhui Zeng, Jianguo Xu

**Affiliations:** ^1^ Department of Neurosurgery, West China Hospital, Sichuan University, Chengdu 610041, PR China; ^2^ National Drug Clinical Trial Institute of West China Second University Hospital, Sichuan University, Chengdu 610041, PR China; ^3^ West China School of Medicine, West China Hospital, Sichuan University, Chengdu 610041, PR China

**Keywords:** neutrophil lymphocyte ratio, intracerebral hemorrhage, prognosis, outcome

## Abstract

The aim of this study was to evaluate the prognostic role of neutrophil lymphocyte ratio (NLR) in patients with spontaneous intracerebral hemorrhage (ICH). PubMed, EMBASE, Web of Knowledge, Cochrane Library and China National Knowledge Infrastructure were searched for potentially relevant literature. The study and patient characteristics were extracted. Odds ratios (ORs) with 95% confidence intervals (CIs) were pooled to estimate the prognostic role of NLR in patients with ICH. Poor functional outcome was defined as modified Rankin Scale≥3. Four studies with 1,720 patients were included. The pooled OR of higher NLR for poor functional outcome at 3 months was 2.74 (95% CI, 1.33-5.65). The pooled OR of higher NLR for death at 3 months was 1.58 (95% CI, 0.44-5.68). Subgroup analysis and sensitivity analysis were also performed. Publication bias was not present. In conclusion, for patients with ICH, higher NLR was associated with poorer functional outcome at 3 months, while higher NLR was not associated with higher risk of death at 3 months.

## INTRODUCTION

Stroke is one of the leading causes of mortality and disability worldwide [1]. Intracerebral hemorrhage (ICH) is the second most common subtype of stroke and represents approximately 10% to 20% of all strokes [2]. ICH is characterized by high rates of mortality and disability, and little effective therapeutic strategies are available currently [3]. Apart from the nature of the hematoma, like volume and position [4–6], some other factors have been identified to be associated with the prognosis of ICH. High blood pressure in the acute phase was widely recognized to be associated with a poor outcome [7], and blood pressure variability has also been shown to predict poor outcome [8]. However the optimal treatment of high blood pressure after ICH is still controversial [9]. Some biochemical parameters, such as cholesterol and ferritin level, were also reported to be biomarkers of outcomes [10, 11]. Besides, it has been suggested that brain imaging parameters may predict outcome in ICH patients, for example, satellite sign and blend sign in the initial CT scan [12, 13].

Recently, a growing body of evidence supports that inflammatory response plays a key role in ICH [14–16]. In this context, some inflammatory markers were suggested to predict outcome in ICH, such as, leukocyte count, neutrophil count, lymphocyte count, neutrophil lymphocyte ratio (NLR), interleukin-6 and C-reactive protein [17]. Among them, NLR was heated studied. And NLR was found to have a prognostic role in various solid tumors and acute ischemic stroke [18, 19]. In recent years, several studies investigating the prognostic role of NLR in ICH were published [20–23]. Some researchers found that higher NLR could predict worse short-term outcome in ICH [20, 22, 23], however, Sun et al. did not come to such a conclusion [21].

Therefore, due to the controversy, we aimed to systematically evaluate the prognostic role of NLR in patients with ICH through performing a meta-analysis.

## RESULTS

### Literature research

**Figure 1:**
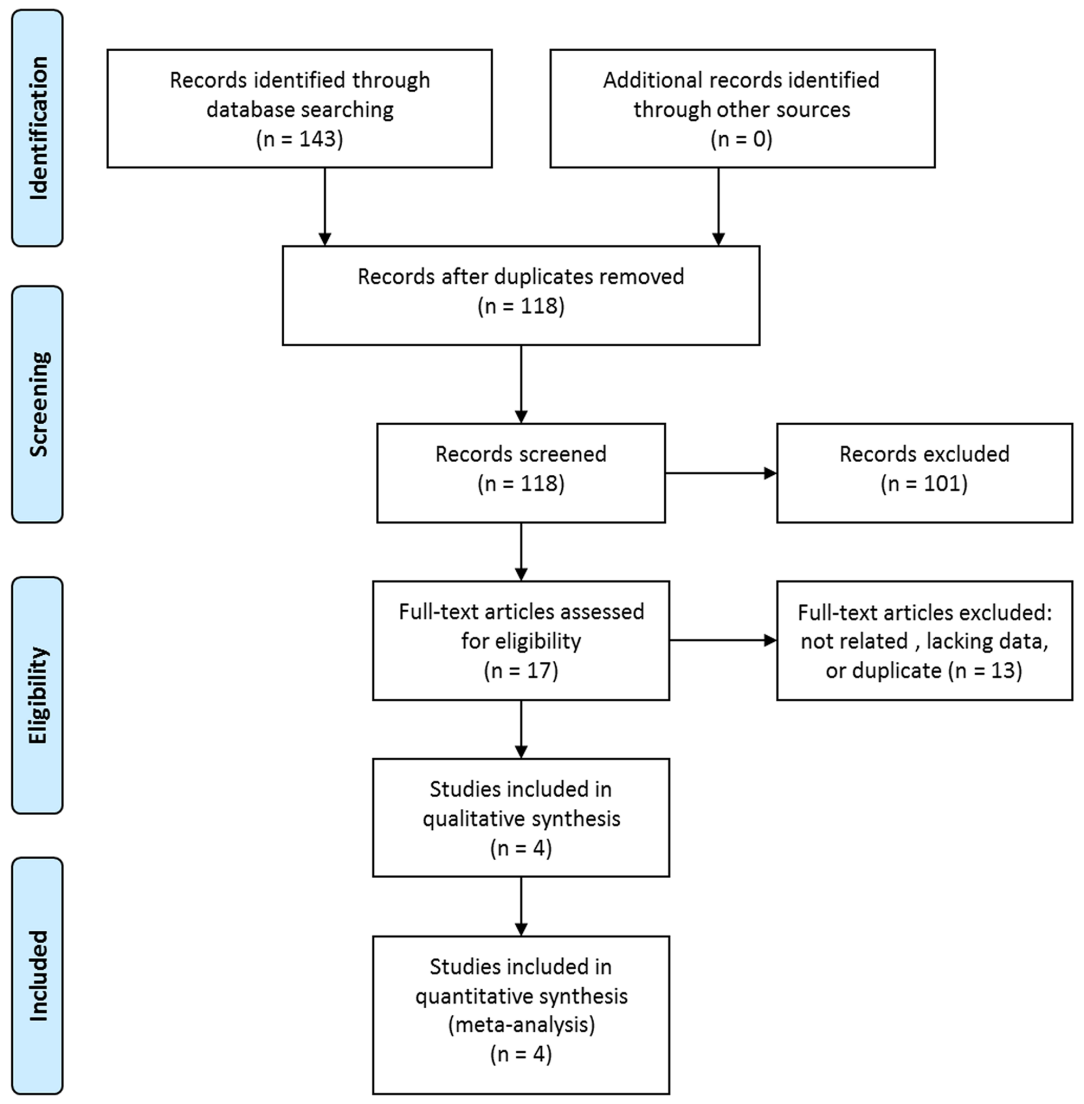
Selection process of studies

The initial literature research retrieved 143 studies. Among them, 25 were duplicated and were removed. After screening for the titles and abstracts, 101 studies were excluded according to the predefined criteria. The rest 17 studies were reviewed in full text and 12 were further excluded due to unrelated or lacking enough data. Two studies were performed in the same institute, and the study periods overlapped [17, 22]. Then, the study not adopting the widely used outcome measure was excluded [17]. Eventually, four articles were included in our study [20–23]. The study selection process was shown in Figure 1.

### Study characteristics

The main characteristics of the 4 included studies were shown in Table 1. All of them were published in the latest two years. Two studies were from China, one was from Germany and the other one was form Italy. A total of 1,720 patients were included (mean 430). The interval between onset and admission was within 24 hours or within 7 days (not reported in one study). Blood samples were collected on admission or on the second morning. The cut-off values of NLR were various. The outcome measures included poor functional outcome at 3 months and death at 3 months. All the ORs were adjusted.

**Table 1:** Characteristics of the included studies

Author	Year	Country	N (F/M)	Age (yrs)	Onset to admission	Sample time	Optimal cut-off value	Outcome measure
Tao	2017	China	336 (120/216)	mean 58.5	≤24 hours	on admission	6.62	death at 3 months
						on admission	6.28	mRS at 3 months
Sun	2017	China	352 (118/234)	mean 64.2	≤7 days	2ed morning	NR	death at 3 months
						2ed morning	NR	mRS at 3 months
Giede-Jeppe	2017	Germany	855 (398/457)	median 72.5 (NLR≥4.66)median 71 (NLR<4.66)	NR	on admission	4.66	death at 3 months
Lattanzi	2016	Italy	177 (114/63)	mean 67.1	≤24 hours	on admission	4.58	mRS at 3 months

N (F/M): number of patients (female/male), yrs: years, NLR: neutrophil lymphocyte ratio, NR: not reported, mRS: modified Rankin Scale.

### Overall analysis

**Figure 2:**
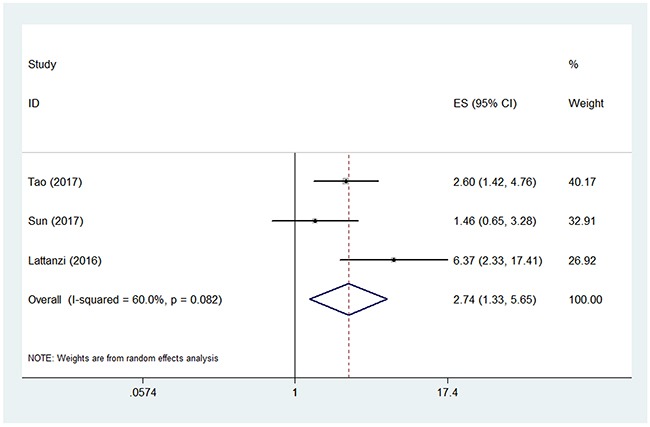
Pooled odds ratio (OR) of higher neutrophil lymphocyte ratio (NLR) for poor functional outcome at 3 months in patients with intracerebral hemorrhage

Three studies used poor functional outcome at 3 months as the outcome measure [20–22]. The pooled OR of the 3 studies was 2.74 (95% CI, 1.33-5.65) (Figure 2), suggesting that higher NLR was associated with higher risk of poor functional outcome at 3 months in patients with ICH. Significant between-study heterogeneity was found (I^2^ = 60.0%, P=0.082). Sensitivity analysis revealed that the study by Lattanzi et al. [22] contributed greatly to the heterogeneity. After removing this study, the heterogeneity shrinked to 20.1% and the pooled OR remained statistically significant (OR 2.11; 95% CI, 1.30-3.43).

**Figure 3:**
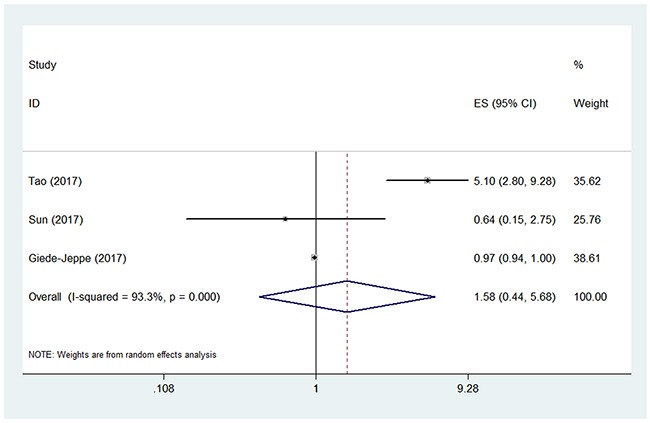
Pooled odds ratio (OR) of higher neutrophil lymphocyte ratio (NLR) for death at 3 months in patients with intracerebral hemorrhage

Three studies used the outcome measure of death at 3 months [20, 21, 23]. The pooled OR of the 3 studies was 1.58 (95% CI, 0.44-5.68) (Figure 3), indicating that higher NLR was not associated with higher risk of death at 3 months in patients with ICH. The between-study heterogeneity was also found (I^2^ = 93.3%, P<0.001). Sensitivity analysis identified that the study by Tao et al. [22] was a significant contributor to the heterogeneity. After excluding this study, the heterogeneity turned sharply to 0%, but the pooled OR was still not statistically significant (OR 0.97; 95% CI, 0.95-1.00).

### Subgroup analysis

As to the three studies examining functional outcome at 3 months, 2 were from China and 1 was from Italy. The pooled OR of the 2 studies from China was 2.11 (95% CI, 1.30-3.43), and the OR of the study from Italy was 6.37 (95% CI, 2.33-17.40). Two of the three studies included patients admitted within 24 hours from onset, and blood samples of the two studies were collected on admission. The pooled OR of the two studies was 3.71 (95% CI, 1.57-8.75). The other study included patients admitted within 7 days from onset and collected blood samples on the second morning, and the OR of this study was 1.46 (95% CI, 0.65-3.28).

As to the three studies examining death at 3 months, 2 were from China and 1 was from Germany. The pooled OR of the 2 studies from China was 2.02 (95% CI, 0.27-15.25), and the OR of the study from Germany was 0.97 (95% CI, 0.95-1.00). Two of the three studies collected blood samples on admission, and the pooled OR was 2.17 (95% CI, 0.43-10.97). The other study collected blood samples on the second morning, and the OR was 0.64 (95% CI, 0.15-2.76).

All the pooled results above were shown in Table 2.

**Table 2:** Summary of meta-analysis results

	N	Model	Pooled OR (95% CI)	P value	Heterogeneity (I^2^, P)	Conclusion	Publication bias
PFO at 3 months							
Total	3	random	2.74 (1.33-5.65)	0.006	60.0%, 0.082	positive	1.000
China	2	fixed	2.11 (1.30-3.43)	0.002	20.1%, 0.263	positive	
Italy	1	—	6.37 (2.33-17.40)	—	—	positive	
Onset to admission ≤ 24h	2	random	3.71 (1.57-8.75)	0.003	55.4%, 0.135	positive	
Onset to admission ≤ 7d	1	—	1.46 (0.65-3.28)	—	—	negative	
Sample time-on admission	2	random	3.71 (1.57-8.75)	0.003	55.4%, 0.135	positive	
Sample time-on 2ed morning	1	—	1.46 (0.65-3.28)	—	—	negative	
Death at 3 months							
Total	3	random	1.58 (0.44-5.68)	0.486	93.3%, <0.001	negative	1.000
China	2	random	2.02 (0.27-15.25)	0.496	85.0%, 0.010	negative	
Germany	1	—	0.97 (0.95-1.00)	—	—	negative	
Sample time-on admission	2	random	2.17 (0.43-10.97)	0.350	96.6%, <0.001	negative	
Sample time-on 2ed morning	1	—	0.64 (0.15-2.76)	—	—	negative	

PFO: poor functional outcome, N: number of included studies, OR: odds ratio, CI: confidence interval.

### Publication bias

**Figure 4:**
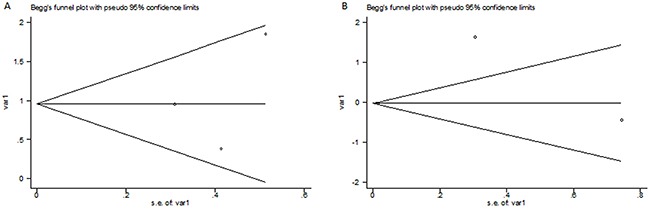
The Begg's publication bias plots of the 3 studies examining functional outcome at 3 months (A) and the 3 studies examining death at 3 months (B) .

No significant publication bias was found as to the 3 studies examining functional outcome at 3 months (p=1.000) or the 3 studies examining death at 3 months (p=1.000). The two Begg's plots of publication bias were shown in Figure 4.

## DISCUSSION

This study intended to assess the prognostic value of NLR in patients with spontaneous ICH. We performed a meta-analysis to summarize the present evidence and four studies were included. To our best knowledge, this is the first meta-analysis on this issue. Our results showed that higher NLR was associated with poorer functional outcome at 3 months in patients with ICH, while higher NLR was not correlated with higher risk of death at 3 months.

Subgroup analyses were also performed to evaluate the value of NLR in predicting functional outcome at 3 months in different settings. The pooled OR of the two studies from China and the OR of the study from Italy were both statistically significant, implying its applicability across countries. However, with a small sample size, caution must be applied. As to the two studies with patients admitted within 24 hours from onset and blood samples collected on admission, the pooled OR was significant, compared to the study with patients admitted within 7 days from onset and blood samples collected on the second morning. Giede-Jeppe et al. [23] found that, in patients with ICH, NLR increased at first, followed by decreasing, and then increased again after admission. The apex was at day 2 (NLR=5.76), and the nadir occurred at day 5 (NLR=4.66) [23]. The NLR also changes over time in patients with acute ischemic stroke [24]. Thus, most investigators used blood samples on admission. A future research direction may be exploring the prognostic role of dynamic NLRs in ICH.

With regards to the three studies examining death at 3 months, the overall OR and ORs in the subgroup were all not significant, suggesting that NLR may not be associated with mortality at 3 months in patients with ICH. Wang et al. [15] assessed the predictive value of NLR in patients with ICH with the endpoint of 30-day mortality, and found that higher NLR exhibited significantly increased 30-day mortality. Therefore, due to the limited study number and significant heterogeneity, more studies are needed to further address the role of NLR in predicting short-term mortality.

Higher NLRs represent both elevated innate immune responses (elevated neutrophils) and decreased adaptive immune responses (decreased lymphocytes) [25]. After ICH, neutrophils are recruited around the hematoma quickly [22, 26]. It has been demonstrated in a mouse model that neutrophil infiltration into the site of hemorrhage induces secondary brain damage [27]. Among patients with ICH, higher peripheral blood neutrophil counts were found to be independently related to poor short-term outcome [20, 22]. Acute central nervous system injury also induces the apoptosis and functional deactivation of lymphocytes [28], which greatly impairs host defense [23]. This may lead to in-hospital infectious complications [22, 23], which are a leading cause of morbidity and mortality in patients with ICH [28]. Giede-Jeppe et al. [23] showed that patients with increased NLR may be associated with higher risk of developing a sepsis and increased short-term mortality.

NLR is a readily accessible and inexpensive marker. In line with some previous studies, our results demonstrated that higher NLR could be a predictor for poor outcome in ICH [20, 22], which could be applied in clinical work. Moreover, as we mentioned above, many other factors were correlated with the outcome of ICH patients. And many of them were controllable, for example, blood pressure and some biochemical parameters. These controllable parameters, as well as inflammatory responses modulation, may represent potential therapeutic targets. Besides, with regards to prognosis, it is promising to perform multidimensional assessment of the known markers and improve prognostic algorithms.

Significant between-study heterogeneities were observed in the meta-analysis. As to the three studies examining functional outcome at 3 months, after excluding the study by Lattanzi et al. [22], the heterogeneity shrinked from 60.0% to 20.1% and the pooled OR remained statistically significant. As to the three studies examining death at 3 months, after removing the study contributed greatly to the heterogeneity [20], the heterogeneity turned form 93.3% to 0.0% but the pooled OR was still not statistically significant. The potential sources of heterogeneity might be from different patient sources, different admission time, different blood collection time and different cut-off values NLR. We did subgroup analyses according to the factors but heterogeneity could not be eliminated by these attempts.

There are several limitations in this meta-analysis. First and foremost, the number of included studies was only four. The number of studies in every subgroup was also limited. These results therefore need to be interpreted with caution. Secondly, some baseline characteristics were different among the studies, such as, the admission time, the blood collection time and the cut-off values NLR. Besides, significant between-study heterogeneities were observed and random effects models were used. Furthermore, no significant publication bias was found in this meta-analysis, but publication bias was a major concern for all meta-analyses and should not be excluded completely.

In conclusion, our results demonstrated that, in patients with ICH, higher NLR was associated with poorer functional outcome at 3 months, while higher NLR was not correlated with higher risk of death at 3 months. As a readily accessible and inexpensive marker, NLR could be applied in the prognosis of ICH with other known markers. The findings also revealed that early intervention regarding inflammatory responses modulation may be a promising therapy in patients with ICH. However, more studies are needed to further address this issue and elucidate the underlining mechanisms.

## MATERIALS AND METHODS

### Search strategy

We followed the developed guidelines for systematic reviews and meta-analyses in performing our meta-analysis [29]. PubMed, EMBASE, Web of Knowledge, Cochrane Library and China National Knowledge Infrastructure (CNKI) were searched for potentially relevant literature (last search ran on May 18th, 2017). The following keywords were used: ‘neutrophil lymphocyte ratio’ AND ‘intracranial hemorrhages’. The reference lists of relevant articles were also screened for additional studies. No language restrictions were used.

### Study selection

Two investigators (J.Z. and S.Z.) independently determined study eligibility, with any disagreements being discussed. Studies were included according to the following inclusion criteria: (1) the patients were diagnosed with spontaneous ICH; (2) blood samples were collected after admission, and NLR was calculated; (3) patients were followed up for survival outcomes or functional outcomes; (4) enough data was reported to estimate the prognostic role of NLR in patients with ICH. Unrelated articles, reviews, case reports, letters, conference abstracts, and studies without enough data were excluded.

### Data extraction

Two researchers (J.Z. and S.Z.) extracted data from the included studies independently, and discrepancies were resolved by consensus. The primary data was odds ratio (OR) with 95% confidence interval (CI) for poorer outcomes. Adjusted OR was extracted if adjusted OR and unadjusted OR were both reported. The study and patient characteristics were also extracted, including first author, publication year, patient source, number of patients, mean or median age of patients, interval between onset and admission, sampling time of blood and the cut-off value of NLR.

### Statistical analysis

Poor functional outcome was defined as major disability or death (modified Rankin Scale [mRS] ≥3) [30]. The logOR and variance, calculated from the OR and 95% CI, were used for aggregation. Forest plots were outlined to estimate the pooled prognostic role of NLR in patients with ICH. The pooled OR was considered statistically significant with the p value less than 0.05 and the 95% CI not overlapping 1. Subgroup analyses were also performed according to patient source, interval between onset and admission, and sampling time of blood. The between-study heterogeneity was assessed, with P<0.10 or I^2^>50% indicating significant heterogeneity [31]. If heterogeneity was present, random effects models were used. Then, sensitivity analysis was performed to assess the contribution of each study to heterogeneity by excluding individual studies one at a time. Publication bias was assessed by Begg's test and p>0.05 was considered that there was no potential publication bias. All the above statistical analyses were performed by STATA 11.0 (STATA Corporation, College Station, TX).
